# ABCD-Trial: study protocol for a randomized, double-blind, placebo-controlled study in patients with *A*myotrophic lateral sclerosis for the evaluation of efficacy and safety of *B*-*C*ell *D*epletion with rituximab (ABCD)

**DOI:** 10.1186/s13063-026-10022-z

**Published:** 2026-10-20

**Authors:** Friederike Ufer, Rosa Rössling, Annika Spottke, Robert Németh, Tamara Schaprian, Nadja Kemmerling-de Coninck, Anna Gamez, Rebecca Sterl, Pia Naumann, Nikolaus Behr, André Maier, Thomas Meyer, Patrick Weydt, Harald Prüss

**Affiliations:** 1 Department of Neurology and Experimental Neurology, Charité –Universitätsmedizin Berlin, Charitéplatz 1, Berlin, 10117 Germany; 2 German Center for Neurodegenerative Diseases E.V. (DZNE) Berlin, Berlin, 10117 Germany; 3 German Center for Neurodegenerative Diseases E.V. (DZNE) Bonn, Bonn, 53127 Germany; 4 Institute of Medical Biometry, Informatics and Epidemiology, University Hospital Bonn, Venusberg-Campus 1, Bonn, 53127 Germany; 5 ALS and Motoneuron Disease Outpatient Clinic, Charité –Universitätsmedizin Berlin, Augustenburger Platz 1, 13353 Berlin, Germany; 6 Motoneuron Disease Outpatient Clinic, Neurozentrum, University Hospital Bonn, Venusberg Campus 1, Bonn, 53127 Germany

**Keywords:** Amyotrophic lateral sclerosis, Rituximab, Autoimmune CNS disorder, Autoantibody, Neurodegeneration

## Abstract

**Background:**

The exact disease-causing mechanisms in ALS remain incompletely understood. A possible cause is inflammation of neurons. Compared to other neurodegenerative diseases, neuroinflammation is pronounced in ALS, presumably leading to disease progression and neuronal death. Patients with ALS can have neuronal autoantibodies and most notably antibody-producing cells in the cerebrospinal fluid. The CD20-binding therapeutic antibody rituximab targets these antibody-producing B-cells. Rituximab is a medication commonly used in neurology. Single patients with ALS have already been treated with rituximab, which was well tolerated. We therefore want to evaluate safety and efficacy of a treatment with rituximab in ALS patients in a double-blind placebo-controlled trial. With this trial we aim for slower disease progression and unveiling disease-causing mechanisms in ALS.

**Methods:**

A total of 52 male and female patients older than 18 years with active sporadic ALS and disease duration of less than 24 months who are on a stable dose of riluzole (50 mg BID) should be included. Patients requiring assisted ventilation of any kind or have a known intolerance to the study medication rituximab are excluded. The study medication rituximab or placebo should be administered over a period of 52 weeks at 4 distinct time points, followed by a follow-up period of 79 weeks with 4 visits. Treatment efficacy should be evaluated using the ALS functioning scale ALSFRS-R-SE at every study visit (except safety visits). Safety will be assessed through regular laboratory analyses, adverse event (AE) and serious adverse event (SAE) monitoring, and CD20+ B-cell kinetics. The primary endpoint is the change in the ALS Functional Rating Scale-Revised (ALSFRS-R-SE) from baseline to week 79. Trial sites will be at Charité/DZNE Berlin and DZNE Bonn.

**Discussion:**

ALS is a rapidly progressive neurodegenerative disease with limited treatment options. Recent preclinical in vivo studies suggest that B-cell-mediated autoimmunity contributes to disease progression. This trial will evaluate whether rituximab, which specifically depletes CD20+ B-cells, can slow ALS progression and provide insights into disease mechanisms.

**Trial registration:**

The trial was registered in the German WHO-recognized primary register Deutsches Register Klinische Studien DRKS (German Clinical Trials Register) on 3/07/2023 under registration number DRKS00030948.

## Administrative information

Note: the numbers in curly brackets in this protocol refer to SPIRIT checklist item numbers. The order of the items has been modified to group similar items (see http://www.equator-network.org/reporting-guidelines/spirit-2013-statement-defining-standard-protocol-items-for-clinical-trials/).

**Table:** 

Title {1}	**ABCD-Trial:** Study protocol for a Randomized, Double-blind, Placebo-controlled Study in Patients with Amyotrophic Lateral Sclerosis for the Evaluation of Efficacy and Safety of B-Cell Depletion with rituximab (ABCD)
Trial registration {2a and 2b}	DRKS, registration number DRKS00030948
Protocol version {3}	Protocol V7.0 13.03.2024
Funding {4}	The clinical trial is funded by the German Federal Ministry of Research, Technology and Space (formally known as: German Federal Ministry of Education and Research, BMBF; funding number 01KG2124). The optional part of the clinical trial including the re-use of clinical data, additional blood sampling and retention of unused CSF for biobanking is funded by the Sponsor.
Author details {5a}	**Friederike Ufer**: Department of Neurology and Experimental Neurology, Charité –Universitätsmedizin Berlin, Charitéplatz 1, 10117 Berlin, Germany **Rosa Rössling:** Department of Neurology and Experimental Neurology, Charité –Universitätsmedizin Berlin, Charitéplatz 1, 10117 Berlin, Germany **Annika Spottke:** German Center for Neurodegenerative Diseases e.V. (DZNE) Bonn, Venusberg-Campus 1/95, 53127 Bonn, Germany **Robert Németh:** Institute of Medical Biometry, Informatics and Epidemiology, University Hospital Bonn, Venusberg-Campus 1, 53127, Bonn, Germany robert.nemeth@imbie.uni-bonn.de **Tamara Schaprian:** German Center for Neurodegenerative Diseases e.V. (DZNE) Bonn, Venusberg-Campus 1/95, 53127 Bonn, Germany **Nadja Kemmerling-de Coninck:** German Center for Neurodegenerative Diseases e.V. (DZNE) Bonn, Venusberg-Campus 1/95, 53127 Bonn, Germany **Anna Gamez:** German Center for Neurodegenerative Diseases e.V. (DZNE) Bonn, Venusberg-Campus 1/95, 53127 Bonn, Germany **Rebecca Sterl:** German Center for Neurodegenerative Diseases e.V. (DZNE) Bonn, Venusberg-Campus 1/95, 53127 Bonn, Germany **Pia Naumann:** German Center for Neurodegenerative Diseases e.V. (DZNE) Bonn, Venusberg-Campus 1/95, 53127 Bonn, Germany **Nikolaus Behr:** Department of Neurology and Experimental Neurology, Charité –Universitätsmedizin Berlin, Charitéplatz 1, 10117 Berlin, Germany **André Maier**: ALS and Motoneuron Disease Outpatient Clinic, Charité –Universitätsmedizin Berlin, Augustenburger Platz 1, 13353 Berlin **Thomas Meyer:** ALS and Motoneuron Disease Outpatient Clinic, Charité –Universitätsmedizin Berlin, Augustenburger Platz 1, 13353 Berlin **Patrick Weydt:** Motoneuron Disease Outpatient Clinic, Neurozentrum, University Hospital Bonn, Venusberg Campus 1, 53127 Bonn **Harald Prüss:** Department of Neurology and Experimental Neurology, Charité –Universitätsmedizin Berlin, Charitéplatz 1, 10117 Berlin, Germany and German Center for Neurodegenerative Diseases e.V. (DZNE) Berlin, 10117 Berlin, Germany
Name and contact information for the trial sponsor {5b}	**Sponsor of the study:** German Center for Neurodegenerative Diseases e. V. (DZNE) Venusberg-Campus 1/99 53127 Bonn Represented by: Prof. Dr. Gabor Petzold Venusberg-Campus 1/99 53127 Bonn Tel: +49–228–43302 811 Fax: +49–228–43302 812 E-Mail: Gabor.Petzold@dzne.de Contact person: Dr. med. Annika Spottke German Center for Neurodegenerative Diseases e. V. (DZNE) Clinical Research Platform (CRP) Venusberg-Campus 1/99 53127 Bonn Tel.: +49 228 43302 820 Fax: +49 228 43302 812
Role of sponsor {5c}	The ABCD trial is an investigator-initiated trial. The financial sponsor is the German Federal Ministry of Research, Technology and Space (formally known as: German Federal Ministry of Education and Research). The financial sponsor had no influence on the study design or development of the trial protocol. The legal sponsor DZNE developed the study protocol and is responsible for the coordination of the trial together with the project lead Harald Prüss.

## Introduction

### Background and rationale {6a}

Amyotrophic lateral sclerosis (ALS) is a devastating, rapidly progressive neurodegenerative disease without effective treatment options [8]. Life expectancy after symptom onset is 2–5 years. The incidence in Germany is at around 3.1/100,000 inhabitants. Although the risk for developing ALS is at 1:400, it ranks among the “orphan diseases” as prevalence is low due the short life expectancy [21].

ALS is characterized by a degeneration of upper and lower motor neurons, which rapidly leads to paralysis, spasticity and muscular atrophy. It ultimately results in death, usually due to respiratory failure. Patients rapidly become dependent in their activities of daily life. Thus, quality of life is often impaired already in early stages of the disease.

Currently, in Germany the only available neuroprotective medication riluzole has an average life-prolonging effect of 2–3 months. Despite manifold research efforts, ALS still is a fatal disease with a striking therapeutic need. Promising results—improved functioning, longer life expectancy and/or quality of life—would substantially change the therapeutic options for the individual patient and the clinical management of ALS. Besides the therapeutic aspect, our trial can markedly add to the understanding of disease development.

#### Presumed mechanisms

The pathophysiological processes behind the progressive motor neuron degeneration in ALS are still incompletely understood. Neuroinflammation in ALS has been recognized as a fundamental disease-promoting mechanism for a long time. At the same time, protein aggregation and the genetic background play a major role in ALS pathophysiology (A). Compared to other neurodegenerative disorders, the inflammatory response in ALS is more pronounced and progression is faster. Thus, inflammation likely contributes to disease progression, aggravates neurodegeneration and leads to the typical spreading of disease [12, 15, 22].

**Fig. 1:**
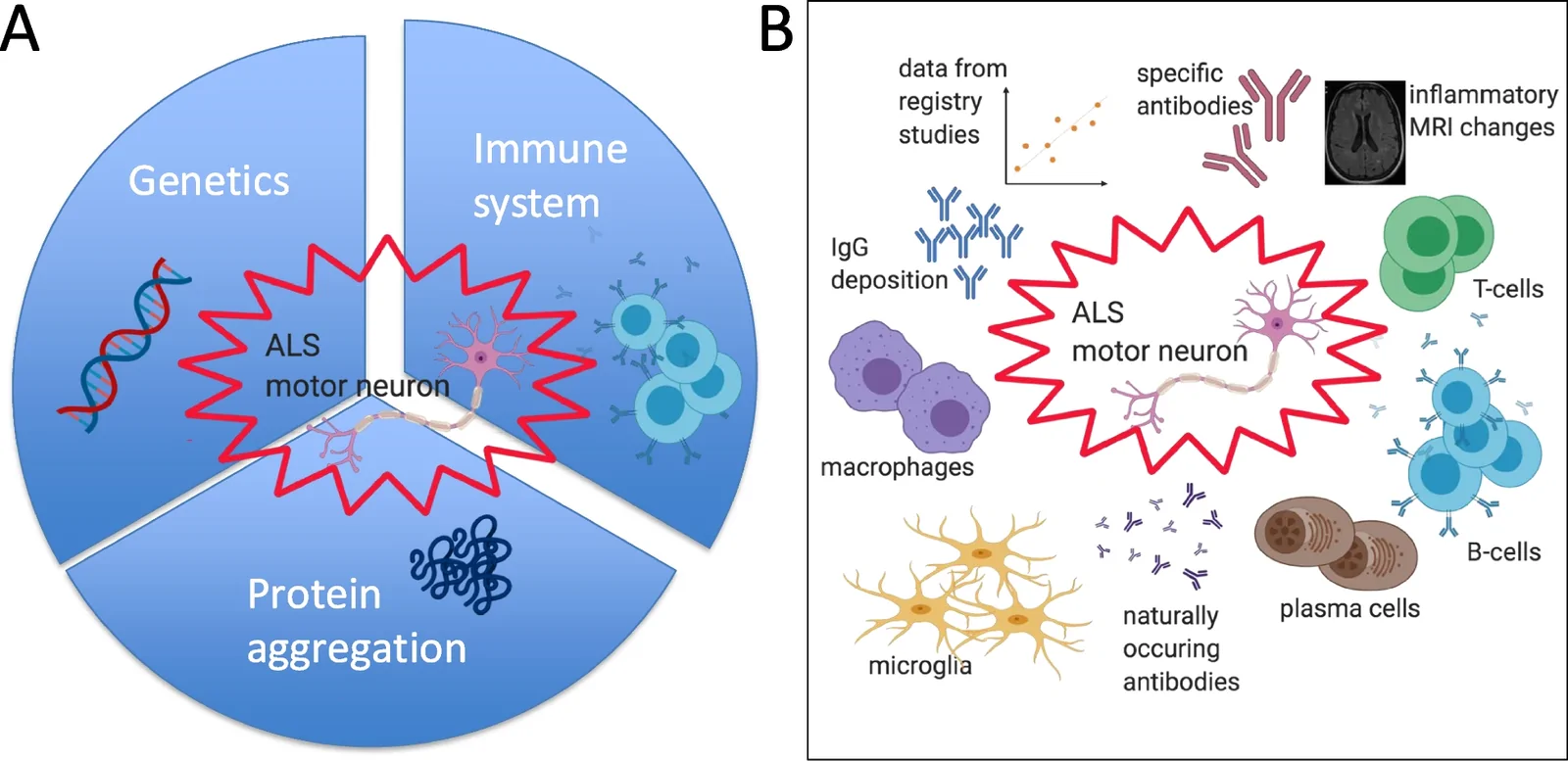
Pathophysiological processes in ALS. **A** Schematic overview of the proposed disease mechanisms contributing to the pathogenesis of ALS. **B** Depicted are immunological abnormalities in ALS covering a wide range of immune cells and inflammatory cascades including humoral autoimmunity

The pathogenic role of the immune system in ALS is supported by both clinical and basic science studies, and involves various types of immune cells (Fig. 1B). For example, two register-based studies showed increased susceptibility to autoimmunity in ALS patients ranging from myasthenia gravis, myositis, Guillain-Barré syndrome to type 1 diabetes and multiple sclerosis (MS) [5]. Cellular dysfunction in ALS involves T- and B-cells. For example, CD8+ T cells that express the ALS-causing superoxide dismutase-1 mutant protein, can kill motor neurons in vitro [18].

Regarding dysfunction of B-cells in ALS, C9orf72 knockout mice, the most common ALS mutation, show a strong autoimmune phenotype with increased activation of B-cell populations [1].

The humoral immune system provides the most direct rationale for B-cell-directed therapy in ALS. IgG deposits localize to motor neurons and to the ventral horns and pyramidal tracts affected in ALS but not to control tissue, and passive transfer of ALS-derived IgG induces motor neuron degeneration in mice, indicating that antibodies can be pathogenic rather than merely reactive [6, 18]. Consistent with an active humoral process, several intracellular-protein autoantibodies are elevated in ALS serum and rise with disease severity, anti-TDP-43 autoantibodies are consistently higher than in controls, and defined targets such as GABA-B receptors have been identified [3, 14, 19]. Because these antibody-mediated mechanisms depend on CD20 + B-cells, they define the therapeutic target this trial addresses by depleting those cells with rituximab. Further evidence for the dysfunctional humoral immune system comes from our personal clinical experience with cerebrospinal fluid (CSF) analyses in ALS patients. Single patients showed signs of neuroinflammation with increased CSF white blood cell counts, increased CSF protein concentrations and positive titers of anti-neuronal autoantibodies.

#### Rationale for selection of investigational medicinal product

Based on these immunological changes in ALS patients with a suspected role of humoral immunity, modulation of B-cell activity may be a specific disease-modifying therapy, e.g., by administration of rituximab, an anti-CD20 antibody. Rituximab effectively depletes B-cells and is safe and well-tolerated in common neuroimmunological indications, such as autoimmune encephalitis, neuromyelitis optica and myasthenia gravis [11]. However, effects are not limited to B-cell-mediated conditions, but also include MS, a primarily T-cell-mediated disease [9]. Hence, B-cell depletion with rituximab targets T-cell activity as well. Compassionate use (“individueller Heilversuch”) of rituximab in single patients with ALS resulted in B-cell depletion and proved to be safe and well tolerated.

Therefore, we conclude that rituximab should be systematically evaluated for a possible effect on disease progression in ALS. As precise targeting of inflammation and at the same time maintaining the protective immune functions is crucial, the specific B-cell depletion by rituximab is promising. This exploratory trial should help to clarify the role of the immune system in ALS. As autoimmunity certainly is only one of several disease-promoting factors, we do not expect to completely stop, but rather to slow disease progression.

We searched the electronic databases PubMed, Cochrane Central/the Cochrane Library, ClinicalTrials.gov, Deutsches Register Klinischer Studien and the International Clinical Trials Search Portal for evidence. Search terms included amyotrophic lateral sclerosis, motor neuron disease or Gehrig’s disease and immune/immunotherapy with no further limits. The latest date of search was 18.03.2024.

We found a very limited number of immune-modulating drugs to have been clinically evaluated in this indication (ClinicalTrials.gov Identifiers: NCT01884571, NCT02469896). The published NIPALS2013 trial (NCT01884571) showed no effect on disease progression using an immunosuppressant from transplantation medicine. The trial showed a dropout rate of >40%, likely because it was including patients even >24 months after disease onset. Furthermore, this study drug did not target antibody-producing cells.

A recent phase-II study (AB0015) used Masitinib, an oral tyrosine kinase inhibitor, as an add-on therapy to riluzole. ALSFRS-R progression rate was significantly slowed in the treatment group compared to the placebo group. This effect might be explained by immunomodulation through targeting of microglia, macrophages and mast cell activity in the central and peripheral nervous system [17].

#### Limitations of previous immunosuppressive trials in ALS

Previous attempts at immunotherapy in ALS using broad immunosuppressive agents such as corticosteroids and cyclophosphamide have failed to show clinical benefit. These failures may be attributable to lack of target specificity and substantial side effects from generalized immunosuppression. The identification of specific autoantibodies and B-cell populations in ALS provides a rationale for more targeted immunotherapy. Rituximab, which specifically depletes CD20+ B-cells while preserving other immune functions, represents a more rational therapeutic candidate based on recent mechanistic insights into B-cell-mediated autoimmunity in ALS.

- Hypothesis:
- We hypothesize that immune mechanisms including low-level anti-neuronal autoantibodies contribute to the pathogenesis of ALS and that depletion of such autoantibodies via B-cell depletion with rituximab leads to reduced progression of ALS symptoms.

### Objectives {7}

#### Primary objective

We hypothesize that B-cell depletion with rituximab compared to placebo will slow ALS disease progression as measured by ALSFRS-R-SE change from baseline to week 79.

#### Secondary and other objectives

Secondary objectives are the further evaluation of ALS-related signs and symptoms as well as of safety and tolerability after the treatment with rituximab. Secondary objectives besides the clinical evaluation of motor deficits include assessment of BMI and the evaluation of the slow vital capacity score.

Further secondary objectives are evaluation of laboratory parameters, evaluation of cognitive deficits and possible changes in the autoantibody repertoire.

Adverse events and serious adverse events are monitored throughout the whole duration of the trial.

### Trial design {8}

ABCD is a multi-center, phase IIa, interventional, randomized, double-blind, placebo-controlled, parallel group trial with an exploratory hypothesis framework. Subjects will be randomized 1:1 either to the active substance (Rituximab) or to placebo stratified by center.

The trial will consist of a screening visit, 4 treatment, 3 safety and 3 follow-up visits with investigations of efficacy and safety.

Visits start with a screening visit that takes place a maximum of 2 weeks before the start of the treatment. The treatment period comprises 4 visits for the administration of Rituximab/placebo. As the first two administrations of Rituximab serve as induction, they need to take place within 2 weeks (± 3 days). The following two infusions will then take place every 6 months (26 weeks ± 14 days). Between the administration of rituximab and 13 weeks ± 14 days after the last IMP administration on-site safety visits are scheduled. As the expected immunosuppressive effect of Rituximab lasts for about 26 weeks, the first follow-up visit will take place 26 weeks ± 14 days after the previous treatment. This visit will constitute the main point to compare treatment effects to baseline. In order to monitor long-term effects on disease progression and possible side effects two further follow-up visits are planned, each 26 weeks ± 14 days apart.

The duration of the clinical trial in the individual subject is 130 weeks as shown in Table 1.

**Table 1:** Schedule of assessments

Assessment/procedure	SCR	Treatment							Follow-up		
		**v1/BL**	**v2**	**v3**	**v4**	**v5**	**v6/EOT**	**v7**	**v8**	**v9**	**v10/EOS**
Informed consent	x										
Demographic data	x										
Medical history	x										
Questioning of special behavior	x										
Previous/concomitant medication	x	x	x	x	x	x	x	x	x	x	x
Questioning of adverse events/clinical events		x	x	x	x	x	x	x	x	x	x
Study diary		x	x	x	x	x	x	x	x	x	x
Clinical examination	x	x	x	x	x	x	x	x	x	x	x
Vital signs	x	x	x	x	x	x	x	x	x	x	x
Blood sample for safety laboratory	x	x	x	x	x	x	x	x	x	x	x
Blood sample for creatin kinase		x	x		x		x		x	x	x
Blood sample for CD20 positive B lymphocytes			x		x		x		x		
Blood sample for albumin, autoantibody and antibody (IgA, IgM, IgG) analysis (serum)		x							x		
Blood sample for safety analysis prior to LP (INR, Quick, aPTT)	x								x		
Blood sample for biobanking (optional)		(x)			(x)		(x)			(x)	(x)
Serology (HIV, HBV, HCV, Mycobacterium tuberculosis)	x										
NPT Assessment (ECAS)		x							x		
ALSFRS-R-SE	x	x	x		x		x		x	x	x
QOL questionnaire SF-36		x	x		x		x		x	x	x
Urine dipstick		x	x	x	x	x	x	x			
Urine pregnancy test	x	x	x		x		x				
Questioning of contraceptive method	x	x	x		x		x				
ECG	x										
Lumbar puncture (CSF standard analyses, cell subtype analysis, antibody (IgA, IgM, IgG) analysis, neuronal autoantibodies)		x							x		
Pulmonary Function Test (Slow vital capacity)	x	x	x		x		x		x	x	x
Eligibility check (inclusion/exclusion criteria)	x	x									
Randomization		x									
Administration of premedication		x	x		x		x				
Administration of rituximab/placebo		x	x		x		x				

*Abbreviations: BL *baseline, *CSF *cerebrospinal fluid, *ECAS *Edinburgh Cognitive and Behavioral ALS Screen, *ECG *electrocardiogram, *EOS *end-of-study visit (v10), *EOT *end-of-treatment visit (v6), *NPT *neuropsychological tests, *(S)AE *(serious) adverse event, *SCR *Screening, *QOL *quality of life

## Methods: participants, interventions, and outcomes

### Study setting {9}

The ABCD trial will be conducted at two academic neurological centers in Germany: the Department of Neurology at Charité Universitätsmedizin Berlin/German Center for Neurodegenerative Diseases (DZNE) Berlin and DZNE Bonn.

### Eligibility criteria {10}

Inclusion criteria are broad in order to include any clinical presentation of sporadic adult-onset ALS. Patients with the diagnosis of clinically definite ALS, or clinically probable ALS based on clinical-neurological examination, electromyography and electroneurography can be included in the study when they meet the following criteria at the screening visit. This will result in a study cohort with comparable or even less comorbidity to MS or encephalitis patients—where rituximab is routinely applied—and thus in a tolerable safety profile:

- Disease duration of sporadic ALS is equal or less than 24 months after symptom onset and symptoms have not progressed to permanent need of assisted ventilation of any kind (including non-invasive ventilation).
- Age ≥ 18 years
- Written consent to participate in the study
- Medication with riluzole at a stable dose of 50 mg BID for ≥ 30 days prior to the screening visit and if possible, throughout the study.
- Slow vital capacity (VC) equal to or more than 75% predicted normal value for gender, height and age at the Screening Visit. The threshold was chosen to ensure adequate baseline respiratory function, thereby minimizing the risk of respiratory complications in the context of immunosuppressive treatment. Preserving sufficient pulmonary reserve is considered essential for patient safety during immunomodulatory interventions.
- Recommended standard vaccination according to STIKO (incl. COVID-19 vaccination). Except for Hepatitis B, Hepatitis B serology will be analyzed at screening as to exclude an acute or underlying infection. In case of missing vaccination, it is mandatory to perform the vaccination parallel to the trial. If necessary, vaccinations should take place four weeks prior to study drug to allow for sufficient vaccination response (dose 1 at least four weeks before first infusion, dose 2 four weeks before third infusion). We will always take current national vaccination recommendations into account.

Patients meeting one of the following criteria cannot participate in the study:

- Dysfunction (other than ALS) that could distort or obscure the diagnosis of ALS.
- Patients with a tracheostomy or ongoing treatment (more than 7 consecutive days in the 4 weeks prior to the screening visit) requiring non-invasive positive pressure ventilation of any kind
- Patients with HIV infections
- Active, severe infections (e.g., tuberculosis, sepsis, and opportunistic infections)
- History of chronically active hepatitis including active or chronic hepatitis B, acute or chronic hepatitis C
- History of a tuberculosis (TB) infection, signs/symptoms of TB or close contact with a
- person with an active TB infection
- Clinically significant infection involving intravenous administration of antibiotics and hospitalization in the 4 weeks prior to the screening visit
- Patients with a serious impairment of the immune system. Patients with other active neuroinflammatory or autoimmune-mediated conditions are excluded to avoid confounding effects on outcome measures and to ensure clean interpretation of treatment effects specifically attributable to ALS pathophysiology.
- Severe heart failure (New York Heart Association Class IV)
- Patients having severe disease in the renal, cardiovascular or hematological system. Patients with significant cardiovascular or renal impairment are excluded due to increased safety risks with rituximab, which requires adequate renal function for drug clearance and cardiac function for volume management during infusions.
- Patients with neutrophils < 1000 cells per μl and/or platelet counts < 75,000 cells per μl
- Any medical condition that, in the opinion of the Investigator, might interfere with the patient’s participation in the trial or poses any added risk for the patient
- Patients with other causes of neuromuscular weakness
- Patients with severe active psychiatric illness
- Patients with a diagnosis of another neurodegenerative disease (e.g., Parkinson disease, Alzheimer’s disease)
- Participation in any other investigational drug study or exposure to an investigational drug within 5 half-lives of the study drug at baseline
- Patients with a history of recurrent or chronic infections or with underlying diseases which may further predispose patients to serious infection
- Sexually active male and female patients of reproductive potential (female patients/female partners of patients less than 12 months postmenopausal) who do not use highly effective contraception methods (pearl index < 1) during and up to 12 months after treatment.
- The patient has used edaravone or sodium phenylbutyrate–taurursodiol in the first week before the baseline visit
- Hypersensitivity to the active substance, mouse proteins or sodium citrate, polysorbate 80
- Known cytokine release syndrome after infusions
- Hypersensitivity to the adjuvant medication (paracetamol, methylprednisolone and dimetindene maleate)
- Pregnancy or lactation

Participant characteristics will be collected and reported in line with the PRO EDI framework (Table 2).

**Table 2:** Pro-EDI: Equity, diversity and inclusion considerations for the ABCD trial. The table summarizes participant characteristics relevant to equity, diversity and inclusion and potential effects of the protocol-defined eligibility criteria on representation and generalizability

Characteristic	Expected participant population	Considerations arising from eligibility criteria
Age	Adults aged ≥ 18 years with sporadic ALS; no upper age limit is specified	No criterion directly excludes participants on the basis of older age. However, older individuals may be more likely to meet exclusion criteria relating to severe cardiovascular, renal, hematological, infectious, or other comorbidities. The respiratory eligibility criterion (slow vital capacity ≥ 75% predicted) may also affect representation across age groups
Sex	Participants of all sexes are eligible	No sex-specific restriction applies apart from reproductive safety requirements. Pregnancy and lactation are exclusion criteria, and sexually active participants of reproductive potential must use highly effective contraception during and for 12 months after treatment
Gender	Participants of all gender identities are eligible; gender identity itself is not an eligibility criterion	Reproductive safety requirements may affect participation and should be applied according to reproductive potential. Predicted vital capacity is determined using the reference equation specified in the protocol, which incorporates relevant physiological variables
Race, ethnicity, and ancestry	No restrictions based on race, ethnicity or ancestry are specified	No eligibility criterion directly restricts participation on these grounds. Representation may nevertheless be influenced by access to specialist ALS services, participating trial centers, diagnostic pathways, and the demographic composition of the populations served by recruiting sites
Socioeconomic status	Participants from all socioeconomic backgrounds are eligible	The requirement to attend study visits may create barriers related to travel, employment, financial resources, mobility, and availability of caregiver or other practical support. These factors may disproportionately affect participants experiencing socioeconomic disadvantage
Geographic location	Participants will be recruited through participating ALS centers as well as through public advertisement, allowing recruitment beyond participating centers	Recruitment through both ALS centers and public advertisement may broaden access to the trial. However, the requirement to attend study visits may still reduce accessibility for individuals living far from trial sites or in areas with limited access to specialist ALS care
Disability and ALS disease stage	The trial targets adults with sporadic ALS of ≤ 24 months' duration from symptom onset who retain relatively preserved respiratory function	Patients with more advanced disease are less likely to be represented because eligibility requires slow vital capacity ≥ 75% and excludes patients with a tracheostomy or prolonged non-invasive positive-pressure ventilation. The findings may therefore be less generalizable to patients with advanced respiratory impairment or later-stage ALS
Capacity and ability to participate	Participants must bei capable to provide written informed consent and be able of attending study visits	People with cognitive impairment that prevents informed consent will not be represented. Requiring attendance at study visits may also reduce participation among people with greater physical disability, limited mobility, limited practical support, or long travel distances
Comorbidity and immune/infectious status	Participants with comorbidities may be included unless protocol-defined conditions pose an additional safety risk or could interfere with trial participation or interpretation	Exclusion of severe cardiovascular, renal, hematological, immunological, infectious, psychiatric, neurological, and other clinically significant conditions is primarily safety-related but is likely to result in a healthier and less multimorbid trial population than the overall ALS population. Exclusion of HIV, specified hepatitis, serious immune impairment, and severe or recurrent infections further limits representation of these groups
Treatment and access to ALS care	Participants are expected to receive standard ALS care, including stable riluzole treatment (50 mg twice daily for ≥ 30 days before screening), where applicable throughout the study	Requiring established riluzole treatment may preferentially select patients already engaged with specialist ALS care. Restrictions on recent edaravone, sodium phenylbutyrate–taurursodiol, and investigational therapies may also affect patients with access to alternative or experimental treatments. Recommended vaccination and timing requirements before rituximab may add logistical constraints

### Who will take informed consent? {26a}

Study physicians with full delegation.

### Additional consent provisions for collection and use of participant data and biological specimens {26b}

There will be optional additional use of serum and CSF samples.

## Interventions

### Explanation for the choice of comparators {6b}

The experimental group of 26 patients will be compared to a group of 26 placebo-controlled patients. In both groups administration of riluzole will be continued as riluzole is the only proven disease-slowing medication and no interactions with rituximab are known. The standard of care riluzole will not be paused in either group, as the study medication is an add-on medication; placebo is an acceptable control.

### Intervention description {11a}

Patients will be randomly allocated to receive rituximab or placebo. The treatment group will receive a total of 4 infusions of 1000 mg rituximab (we will use the biosimilar RIXATHON**®**, Hexal) in solution of 0.9% sodium chloride. The placebo consists of 0.9% sterile saline solution prepared in identical infusion bags to maintain blinding. Study pharmacists prepare both rituximab and placebo infusions, and clinical staff administering infusions are blinded to treatment allocation. Placebo infusions follow the same volume and infusion rate as rituximab. To preserve blinding and minimize infusion reactions, all participants (both treatment arms) receive standardized premedication (see below).

Infusion time will be approximately 4 h. Both groups will be treated with intravenous infusions of 1000 mg paracetamol, 100 mg methylprednisolone and 4 mg dimetindene maleate as premedication 30 min prior to the study medication. All visits will be hold by study physicians. Administration of study medication/placebo can be carried out after exclusion of infection by history, physical examination and blood control of leukocytes and CRP as well as a urine sample. All safety assessments follow institutional standards. Patients will be seen in the outpatient department of the respective clinic or at the clinical trial unit of the DZNE. The treatment visits 1 and 2 will take place only two weeks apart; followed by treatment visits 4 and 6 every 26 weeks. The 26-week dosing interval is justified by the expected timing of B-cell repopulation following 1000 mg dosing, ensuring sustained B-cell depletion while minimizing unnecessary exposure. Three follow-up visits are planned starting 26 weeks after the last treatment visit and continuing every 26 weeks. Additional safety visits will take place after 15 weeks and 43 weeks during the treatment period and in week 71 during follow-up.

### Criteria for discontinuing or modifying allocated interventions {11b}

Subjects are subsequently excluded if they withdraw their consent to participate in the study. The patient has the right to withdraw his/her consent to participate in the study at any time without giving reasons and without any negative consequences for further medical care. The patient is excluded if he/she meets an exclusion criterion in the screening visit or misses too many visits according to the investigator. Additionally, the investigator can decide that the treatment of a subject must be discontinued due to an adverse event or because it poses a risk to the subject or cannot be tolerated from an ethical point of view. In case of pregnancy of the participating patient the IMP administration must be discontinued.

### Strategies to improve adherence to interventions {11c}

As for most of the eligible participants, there is no other disease-modifying therapy available, we expect participants highly motivated to participate throughout the study.

### Relevant concomitant care permitted or prohibited during the trial {11d}

Concomitant treatment with the standard of care drug riluzole at a stable dose of 50 mg BID for ≥ 30 days prior to screening is mandatory and must be kept, if possible, throughout the study. Premedication must be administered before every infusion of rituximab/placebo (for details see above). Edaravone should not have been used in the week before the baseline visit. Vaccination with live virus vaccines is prohibited during the clinical trial, since the safety of immunization with live viral vaccines following rituximab therapy has not been studied so far.

### Provisions for post-trial care {30}

In case of premature termination of treatment by the patient or the study physician the follow-up visits (visit 8, 9, 10) should be attended at the same interval as planned (i.e., starting 26 weeks after the last IMP administration) for safety reasons. If that is not possible or favored by the subject, at least one follow-up visit 26 weeks after the last infusion of the study medication (according to visit 8) is strongly recommended.

After completion of the trial, the subjects will continue receiving standard of care treatment according to current national guidelines.

Any decisions regarding post-trial treatment will be made independently by the treating physician based on individual patient circumstances, available evidence, and clinical judgement. Such decisions are entirely separate from the trial protocol and will not be influenced by the study team. Patients who may benefit from continued treatment will be informed about the off-label status of rituximab in ALS and referred to their treating neurologist for independent clinical decision-making. Usually, in patients with autoimmune encephalitis rituximab is discontinued after 4–5 infusions.

### Outcomes {12}

The primary endpoint is the ALSFRS-R-SE change from baseline to week 79 (Maier, 2022). The ALSFRS-R-SE (Self-Evaluation) is a modified version of the ALS Functional Rating Scale–Revised in which items are completed by the patient rather than assessed by a clinician. Symptom progression should be evaluated with the ALSFRS-R-SE and its change from baseline (first treatment visit) to 79 weeks after administration of the first dose of Rituximab. The performance of the ALSFRS-R-SE is expected to be similar to the ALSFRS-R with respect to the clinical relevance.

As this is an exploratory pilot, a clinically encouraging signal is defined—independent of statistical significance—as a descriptively slowed ALSFRS-R-SE decline approaching the ALSFRS-R MCID range (~2–4 points or ~20–25% slowing), a delay in time to key milestones (loss of ambulation, initiation of NIV), or sustained stabilization over a defined interval.

Secondary endpoints will be analyzed in an exploratory manner without hierarchical testing or adjustment for multiplicity, given the pilot nature of this trial. Secondary objectives are grouped into clinical, physiological, laboratory, and patient-reported outcome measures. Clinical outcomes include the assessment of motor symptom progression using the ALSFRS-R-SE from baseline to weeks 3, 27, 53, 105, and 130 (visits 2, 4, 6, 9, and 10), as well as tracheostomy-free survival at week 79.

Physiological outcomes comprise changes in body mass index (BMI) assessed longitudinally during the study and pulmonary function measured by slow vital capacity from baseline to week 79. Symptom progression measured with the ALSFRS-R-SE as well as BMI development is the most relevant outcome measure for the individual patient.

Laboratory outcomes include serum and cerebrospinal fluid analyses of infection markers, B-cell counts, and potential changes in the autoantibody repertoire.

CD20+ B-cell depletion kinetics will be related to clinical outcomes in exploratory correlation analyses, and these biomarker analyses will also be used to probe for potential responder subgroups. Failure to achieve or maintain B-cell depletion could be interpreted as a negative mechanistic result even if clinical trends appear favorable, as any clinical signal without target engagement cannot be attributed to the intended mechanism.

Patient-reported and cognitive outcomes include the assessment of cognitive function using the Edinburgh Cognitive and Behavioural ALS Screen (ECAS) and quality-of-life questionnaires, administered at baseline and at week 79 after the first dose of rituximab. ECAS was selected because it is validated for ALS populations across multiple languages and is sensitive to frontotemporal dysfunction commonly observed in ALS patients.

All adverse events and serious adverse events are monitored throughout the entire duration of the trial.

### Participant timeline {13}

**Fig. 2:**
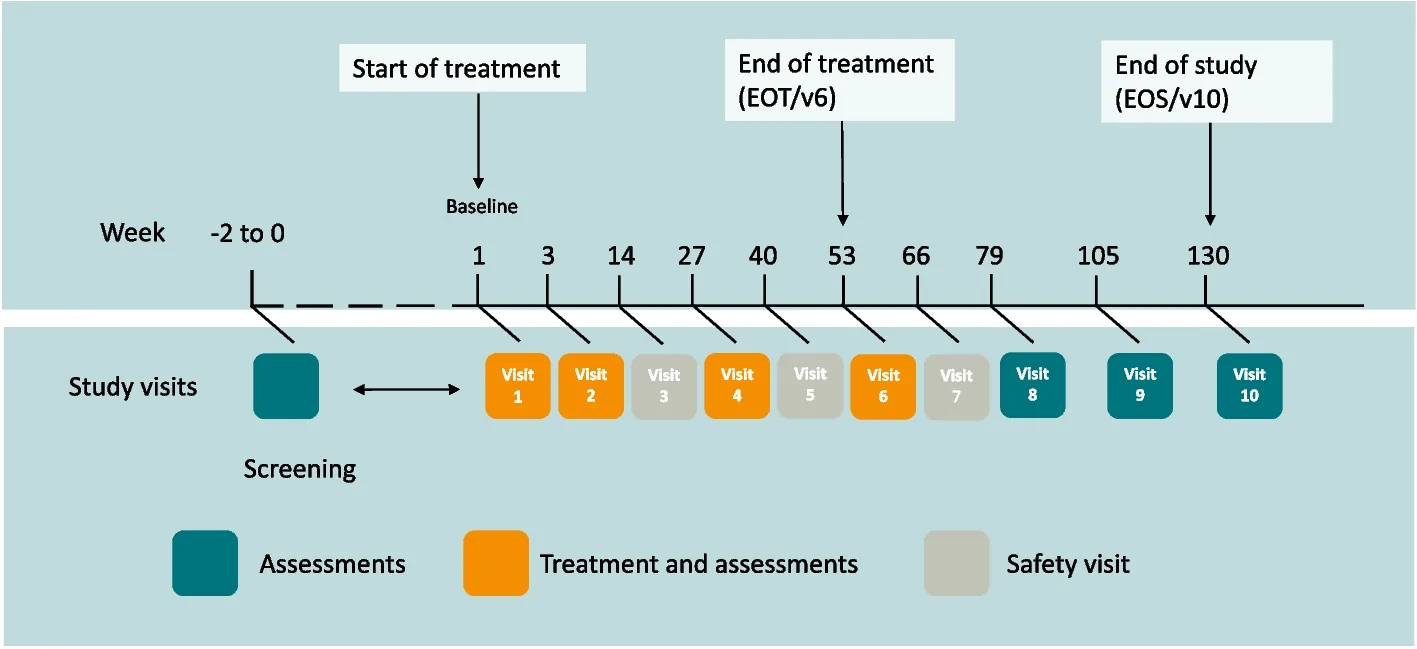
Overview of the individualized treatment plan and study procedures for each study participant

The study will consist of a 53-week double-blind treatment period, which will be followed by a 77 weeks follow-up period. Participants will be randomized in a 1:1 ratio to receive rituximab or placebo. We plan to recruit 52 patients over a 24-month period (approximately 2–3 patients per month across both sites). Each patient will participate for 130 weeks (53 weeks of treatment followed by 77 weeks of follow-up (Fig. 2)). The total study duration, from first patient enrolled to last patient completed, is estimated at 54 months (24 months of recruitment plus 30 months of per-patient participation). Study visits will be conducted on week 1, week 3, week 27, and week 53 during the treatment period. Follow-up visits will be performed at week 79, week 105 and week 130 including the sampling of blood, urine, ALSFRS-R-SE and documentation of AEs. Additional safety visits will take place on week 14 and 40 during the treatment period and on week 66 during follow-up (Fig. 2).

Time schedule of the study:

Inclusion of first patient, first patient first visit (FPFV): June 2024

Inclusion of last patient, last patient first visit (LPFV): June 2026

Completion of last patient (LPLV): December 2028

Closure of the database: January 2029

Final report: August 2029

### Sample size {14}

A total of 104 patients will be assessed for eligibility, of whom 52 will be allocated to the pilot trial. Based on an anticipated drop-out rate of approximately 20%, around 40 patients are expected to be included in the final analysis.

#### Rationale for pilot study design

The present study is designed as a pilot trial with non-confirmatory objectives. As no preliminary data are available on the ALSFRS-R-SE score in patients with amyotrophic lateral sclerosis (ALS) treated with rituximab, a conventional power calculation for formal hypothesis testing is not feasible. Consequently, the study is not powered to draw confirmatory conclusions regarding treatment efficacy.

The primary objectives of the pilot study are to assess feasibility and to estimate key statistical parameters required for the planning of a subsequent confirmatory trial, in particular the variability (standard deviation) and the preliminary effect size of the ALSFRS-R-SE estimand.

#### Sample size determination for the pilot study

The sample size of the pilot study was determined based on methodological recommendations for pilot trials, focusing on the precision of variance estimation rather than statistical power. Using the upper confidence limit approach for the standard deviation as implemented in PASS software (version 19; procedure: Sample Size of a Pilot Study by Upper Confidence Limit of SD), an overall sample size of 40 evaluable patients was estimated to provide sufficient precision for planning a future confirmatory trial under the assumptions of a two-sided significance level of *α* = 0.05, a target power of 80%, a mean difference of *D* = 2.5 points, and standard deviation value of 6.6.

Allowing for an anticipated drop-out rate of approximately 20%, this results in an envisaged overall sample size of *N* = 52 patients to be allocated to the pilot trial, corresponding to an estimable standard deviation of approximately 6.6.

#### Link to the planned confirmatory trial

The variance and effect size estimates obtained from the pilot study will be used to inform the sample size calculation and design assumptions of a subsequent randomized confirmatory trial. For illustration, assuming a two-sided t-test, a mean difference of *D* = 2.5 in the ALSFRS-R-SE score, and standard deviation value of 7 total sample sizes of 252 patients (1:1 allocation) would be required to achieve 80% power at a significance level of α = 0.05. Accounting for an anticipated drop-out rate of approximately 20%, this would correspond to total sample sizes of approximately 316 patients, respectively.

The assumed values for the ALSFRS-R-SE score are based on analogy with the ALSFRS-R and its performance in previous ALS clinical trials reported in the literature [4, 10, 13].

Progression to a confirmatory phase III trial will be guided by prespecified go/no-go considerations combining clinical, biomarker, and feasibility signals. A descriptively favorable clinical signal (a slowing of ALSFRS-R-SE decline approaching the ALSFRS-R MCID range, as defined above) together with evidence of target engagement (sustained CD20+ B-cell depletion) would support continuation, whereas absence of biological effect would argue against it regardless of apparent clinical trends. Feasibility metrics—recruitment rate, treatment adherence and the observed safety profile—will be treated as formal go/no-go criteria for the confirmatory trial. The variance and effect-size estimates from this pilot will concretely inform the sample size, allocation, and endpoint selection of the confirmatory study, as illustrated above.

#### Compliance/rate of loss to follow-up

In a recent phase-II trial, mentioned earlier already, that assessed the safety and efficacy of masitinib in a total of 394 ALS patients, only 1 patient needed to be excluded for poor compliance, and 2 patients were lost to follow-up [17]. Similar numbers were seen in a phase-III trial assessing safety and efficacy of edaravone in ALS patients: out of 137 eligible patients a total of 10 patients discontinued treatment [20]. These examples underline the strong adherence to therapy in ALS patients.

### Recruitment {15}

In Germany, every year around 2000 patients are diagnosed with ALS. Two hundred fifty new patients present every year in the ALS outpatient clinic of the Charité and approximately 150 patients in the motoneuron outpatient clinic of the university hospital Bonn. The majority of new patients will meet the inclusion criteria and are therefore eligible for participation. Both ALS centres regularly participate in pharmaceutical studies and are trained in study participation. Clinical experience shows that ALS patients’ willingness to take part in treatment studies is extremely high, further indicating that patient recruitment for this trial will be successful.

**Table:** 

Trial site	Number of eligible patients/year
Charité Berlin	50
University Hospital Bonn	61

## Assignment of interventions: allocation

### Sequence generation {16a}

Subjects eligible for treatment will be randomized after final check of in- and exclusion criteria before the first administration. The patients will be randomized into two equal study arms (at each of the two sites) with a total amount of 52 patients. The randomization is stratified by centre to achieve a sufficiently frequent allocation of used therapy arms in both centres. Additional stratification by baseline ALSFRS-R-SE slope, site of onset (bulbar versus limb) or sex was not implemented because the small total sample size (*n* = 52) would leave individual strata too sparsely populated to permit balanced allocation, and finer stratification carries a risk of incomplete or unfilled strata that can itself introduce imbalance in a trial of this size. To mitigate the resulting risk of chance imbalance, key baseline prognostic variables (baseline ALSFRS-R-SE and slope, site of onset, sex, age and disease duration) will be compared descriptively between treatment groups, and any variable found to be strongly imbalanced will be explored as a covariate in sensitivity analyses of the primary endpoint.

### Concealment mechanism {16b}

After checking the in- and exclusion criteria by study physicians a randomization number will be requested by the pharmacy. The authorized staff will assign in chronological order a randomization number. The study medication will be delivered blinded so that investigators and patients will not be aware of the assignment to placebo or rituximab treatment. Preparation of the blinded infusions will be done by the manufacturing company/pharmacy. It is not possible to distinguish between verum and placebo infusions; the labeling of both infusion bags will be identical.

### Implementation {16c}

The allocation sequence is generated by the SZB (Studienzentrale Bonn). The generated randomization lists will only be accessible to authorized staff (Safety Management (SZB) and Pharmacy (Medios Solution Bonn GmbH, formally known as Rheinische Compounding and Apotheke Charité Berlin).

## Assignment of interventions: blinding

### Who will be blinded {17a}

The study has a double-blind design. All investigator and trial team members will be kept blind to the medication throughout the trial unless medical conditions require unblinding, e.g., in case of SUSARs (suspected unexpected serious adverse reaction). Blinding of the study medication will be carried out by manufacturing companies, the hospital pharmacy of Charité Berlin and the Medios Solution Bonn GmbH (formerly known as Rheinische Compounding). SZB will create the randomization lists and the emergency envelopes for both sites.

Laboratory staff performing biomarker analyses will be blinded to treatment allocation and will receive only coded samples with no identifying information about treatment assignment.

### Procedure for unblinding if needed {17b}

Blinding of treatment assignment is critical to the integrity of this clinical study. In the event of a medical emergency or pregnancy in an individual participant in which knowledge of the investigational product is critical to the subject’s management, the blind for that participant may be broken by the Investigator. The subject’s safety takes priority over any other considerations in determining if a treatment assignment should be unblinded.

Each site receives a total of 52 emergency envelopes to account for the possibilities in patient distribution between the centers.

Unblinding of the administered study medication can be done by the investigator with the help of these emergency envelopes in case of emergency. Before breaking the blind of an individual participant's treatment, the investigator should determine that the unblinded information is necessary, i.e., that it will alter the participant's immediate management. In many cases, particularly when the emergency is clearly not related to the investigational product, the problem may be properly managed by assuming that the participant is receiving active product. When the blind was broken, the Sponsor and project leader must be informed immediately. Unblinding must be documented properly. The investigator must document the reasons for unblinding in the subject’s source documents. The investigator is strongly advised not to divulge the subject’s treatment assignment to any individual not directly involved in managing the medical emergency, or to personnel involved with the analysis and conduct of the study. The investigator can contact the Sponsor or project leader to discuss such situations.

## Data collection and management

### Plans for assessment and collection of outcomes {18a}

All trial-relevant data will be recorded in the eCRF by the investigator or study assistant.

The patient files, laboratory results, ECG printouts, questionnaires, imaging media and X-ray images are to be evaluated as source data.

The additional sheets and patient files must be completed in full and signed by the principal investigator personally. In the event of corrections, the original entry must be crossed out so that it is still legible. The correction must be dated and provided with an abbreviation.

### Plans to promote participant retention and complete follow-up {18b}

To promote participant retention and complete follow-up, patients are monitored closely and additionally supervised by our outpatient clinic. Patients will be educated on the importance of follow-up for safety reasons. If it is unclear why patients are lost to follow-up, study personnel will try to contact the patients to elucidate the reasons for it and, if possible, motivate them to continue.

### Data management {19}

The DZNE creates a specific eCFR for the study that features CFR 21 Part 11 and GCP compliant data entry and which will reliably and centrally store research data and information and provide significant scientific added value via quality controls. The central data management is responsible for the technical set-up of the study, for implementation of study specifications based on the study protocol, for carrying out plausibility checks, and for data cleaning, archiving and export. At the end of the study the clean data records will be provided to the study statistician for analysis.

Essential data of the clinical trial will be archived for at least 25 years according to Regulation (EU) No. 536/2014. The data will be stored in a specific section of the research drive for this study. All shared data will be pseudonymized or anonymized; no patient identifiable data will be made available at any time. Data dictionaries will be shared.

### Confidentiality {27}

The provisions of the Federal Data Protection Act and General Data Protection Regulation are observed in the trial. It is ensured that all research materials and data are adequately pseudonymized prior to scientific utilization in accordance with data protection regulations.

During the clinical trial, medical findings and personal information are collected from the subjects and written down in their personal files or stored electronically at the trial centre. The data important for the clinical trial are also stored in pseudonymized form, evaluated and, if necessary, passed on. The subjects’ surname, first name, date of birth, contact details (postal address, telephone number, email address), i.e., “identification data” (IDAT) are stored in a database that is separate from the study data at a data trustee. The collected data (“medical data” (MDAT)) are pseudonymized and stored in a database that is separate from the IDAT and secured against unauthorized access. “Pseudonymized” means that no names or initials are used, but only a number and/or letter code, possibly including the year of birth.

All persons who have insight into the data of the subjects are obliged to protect data protection and are subject to a confidentiality requirement. The data are secured against unauthorized access. Decoding takes place only under the conditions prescribed by law, particularly in case of medical emergency. The personal data will be deleted after 25 years.

As part of the subject information, the subjects are informed about the transfer of their pseudonymized data within the scope of the documentation and notification obligations according to CTR to the recipients named there (see subject information). Persons who do not consent to the disclosure cannot be included in the clinical trial.

### Plans for collection, laboratory evaluation and storage of biological specimens for genetic or molecular analysis in this trial/future use {33}

CSF Processing: CSF samples will put on ice immediately and will be processed within 15 min of collection. Samples are centrifuged at 2000 g for 10 min at 4 °C to remove cells. Supernatant is aliquoted into 500 μL cryovials (minimum 3 aliquots per sample) and immediately frozen at − 80 °C for long-term storage.

Blood Processing: Blood samples for serum analysis are allowed to clot for 30 min at room temperature, then centrifuged at 2000 g for 10 min. Serum is aliquoted into 500 μL cryovials and frozen at − 80 °C.

Central vs. Local Laboratory:

- B-cell flow cytometry (CD20 + counts): Performed at local site laboratories within 4 h of collection.
- Autoantibody analyses: Performed at central laboratory (Charité Berlin) on batched frozen samples.
- Routine safety labs (CBC, chemistry): Performed at local clinical laboratories per institutional standards.

Sample analyses will be performed by local laboratories and Experimental Neurology at Charité Berlin. In addition, optional donation of serum and CSF samples may be collected for future investigations. These samples will be stored by the Sponsor in the Biobank Clinical Research at DZNE in Bonn and made available for research purposes on request.

## Statistical methods

### Statistical methods for primary and secondary outcomes {20a}

A descriptive summary analysis is planned for all endpoints and will include mean, minimum, maximum, standard deviation, median and the IQR (interquartile range) for continuous variables and counts and percentages for categorical variables for all randomized patients. The number of patients who were randomized, discontinued or completed the study will be summarized by treatment groups.

Full details of the analysis will be given in the statistical analysis plan. All data will be analyzed by using SAS version 9.4 (or later).

The secondary survival-type endpoints will be analyzed by means of Cox proportional hazard model including at least the effects of treatment and center.

The primary estimand will follow a treatment policy strategy and is defined to descriptively characterize treatment effects in this pilot study using an exploratory MMRM approach.

The modified intention-to-treat (mITT) population will be used for the primary analysis and will comprise all randomized patients who receive at least one dose of double-blind study medication. The individual endpoint is the ALSFRS-R-SE score. Analyses will descriptively characterise changes in ALSFRS-R-SE from baseline to the visit in week 79 between the two treatment groups.

Treatment effects will be estimated using an exploratory mixed model for repeated measures (MMRM), based on all available post-baseline observations (assuming missingness at random (MAR) conditional on the variables included in the model) applying appropriate contrasts to estimate differences in mean change over time. The model will include fixed effects for treatment, center and visit, as well as the repeated effect of time (four visits). All model-based estimates will be interpreted descriptively and for hypothesis-generating purposes only.

As an additional exploratory analysis, a model-based estimator using an analysis of variance (ANOVA) framework will be applied to individual change scores from baseline to week 79. A further sensitivity analysis will use an exploratory MMRM approach combined with imputation methods for missing data, with the aim of descriptively assessing the impact of missingness on the estimated treatment effects. Since missing outcome values may result from intercurrent events such as death, the MAR assumption may be violated. Hence, for sensitivity analysis, no single-value imputation (e.g., last observation carried forward) will be applied. Given the potential association between functional decline, treatment discontinuation, and mortality, sensitivity analyses will assess the robustness of the primary MMRM results to deviations from the MAR assumption, including MNAR/pattern-mixture and tipping-point analyses. Additionally, the reasons for missing outcome values will be listed. Details on the handling of missing values and the deviation from the MAR assumption will be specified in the SAP.

A secondary estimand following a hypothetical strategy will be defined for a per-protocol (PP) population, comprising patients treated according to the protocol for the full study duration without relevant intercurrent events. The same exploratory estimators as for the primary estimand will be applied.

Estimated differences will be interpreted descriptively with reference to mean and median changes in ALSFRS-R-SE and discussed in relation to the Minimal Clinically Important Difference (MCID).

For the ALSFRS‑R, published estimates of the minimal clinically important difference (MCID/MID) range from approximately 2 to 4 points on the total score (e.g., patient‑reported MID ~3.24 points over follow‑up periods in Fournier et al. [7]) and clinician‑defined meaningful change thresholds (≈20–25% slowing of decline in Castrillo-Viguera et al. [2] in the literature. As no MCID has been formally established for the ALSFRS‑R‑SE, these values are applied by analogy. Accordingly, estimated differences in ALSFRS‑R‑SE observed in this pilot study will be interpreted descriptively in relation to this range, acknowledging the exploratory nature of this approach and the absence of a validated MCID for the SE version.

Secondary endpoints will be summarized using descriptive statistics only. Missing values will generally not be imputed.

Exploratory analyses of survival-type endpoints will be performed using Cox proportional hazards models including at least treatment and center as covariates. Results will be interpreted descriptively.

To explore the robustness of findings, results obtained from the mITT and PP populations will be descriptively compared.

The AEs will be coded using the most recent version of the Medical Dictionary for Regulatory Activities (MedDRA) coding. A treatment-emergent adverse event (TEAE) is defined as an event that has onset date on or after the first day of exposure to randomized treatment and no later than re-normalization of CD20+ cells has occurred. We have defined CD20+ B-cell re-normalization as return to pre-treatment baseline levels ± 20%. If the event has onset date before the first day of exposure on randomized treatment and increases in severity during the treatment period and until re-normalization of CD20+ cells, then this event should also be considered as a TEAE. TEAEs are summarized descriptively, whereas non-treatment-emergent AEs are presented in listings. TEAE data will be displayed in terms of the number of subjects with at least one event (*N*), the percentage of subjects with at least one event.

### Interim analyses {21b}

No interim analysis is planned.

### Methods for additional analyses (e.g., subgroup analyses) {20b}

No secondary analyses are planned.

### Methods in analysis to handle protocol non-adherence and any statistical methods to handle missing data {20c}

#### Analysis population

All analyses will use the following analysis sets:

- Modified intention-to-treat (mITT) set,
- Per-protocol (PP) set,
- Safety (SAF) set.

The mITT set includes all randomized patients who received at least one dose of the double-blind study medication.

The PP set includes all randomized patients who were treated according to the study protocol without major protocol violations. Major protocol violations are defined according to the following list:

- Violation of major eligibility criteria, to be determined during blind review meeting
- Missing of one treatment visit (administration within 7 days after the scheduled visit will not lead to a major protocol violation)

The SAF set includes all randomized patients who received at least one dose of the study medication and for whom at least one safety blood analysis was performed, and a respective value was reported.

Before database closure a blind review meeting takes place in which the allocation of disputable patients to the populations will be discussed and decided.

Missing or incomplete data for survival is managed by censored data analyses. To control robustness of analysis mITT- and PP-results will be compared. Missing data for continuous endpoints, including ALSFRS-R-SE, will be handled using multiple imputation under the missing-at-random assumption. The imputation model will include treatment group, baseline ALSFRS-R-SE score, and all variables used in the primary analysis model. Sensitivity analyses using complete-case analyses will be performed to assess the robustness of the results.

The estimated difference will be interpreted under the aspect of discussing the mean/median values of differences of ALSFRS-R-SE belonging to the MCID of the ALSFRS-R.

### Plans to give access to the full protocol, participant-level data, and statistical code {31c}

Access to the full protocol and the Clinical Study report is given by the Clinical Trials Information System (CTIS) as the online system for regulatory submission, authorization and supervision of clinical trials in the European Union.

## Oversight and monitoring

### Composition of the coordinating center and trial steering committee {5d}

Although no formal steering committee has been established, overall trial oversight and management are provided by the Project leader Prof. Harald Prüß together with the medical coordinator Anna-Maria Weiß as well as the project management of DZNE, Dr. med. Annika Spottke. They are responsible for overseeing trial conduct and progress, coordinating the participating study sites, monitoring recruitment and protocol adherence, and addressing relevant operational issues throughout the trial. Trial progress is regularly reviewed and discussed in meetings with the participating study sites.

### Composition of the data monitoring committee, its role, and reporting structure {21a}

A data safety and monitoring board (DSMB) will be formed to ensure the participant’s safety. The DSMB is a group of independent experts external to the study. The DSMB will have a meeting or telephone conference at least once per year for safety analysis. Additional times may be added if warranted by safety considerations (e.g., accumulation of SAE or AE). Before the meeting the members will receive a list of all reported AEs, SAEs and relevant safety related data. The DSMB will evaluate the safety data and the progress of the study and will make recommendations of the continuation, modification or termination of the clinical trial in accordance with the tasks documented in the DSMB manual. If there is for example a suspect accumulation of SAE in any treatment arm, the coordinating investigator decides in agreement with the DSMB and the responsible statistician on the continuation of the trial. In weighing any safety signal, the DSMB will explicitly balance the treatment-related risk against the severity and rapid progression of ALS, recognizing that a degree of infection risk is considered acceptable in this early-stage, relatively preserved population given the disease’s high mortality and the absence of effective alternatives. e’s high mortality and the absence of effective alternatives.

The Sponsor/project leader will inform investigators of all SUSARs in unblinded form including all relevant further information within the periods set by the competent authority.

The Sponsor/project leader will inform all investigators of any change of information concerning the scientific documents of the trial (SmPC, Investigator’s Brochure, risk–benefit ratio).

### Adverse event reporting and harms {22}

The principal investigator ensures that all persons involved in the treatment of trial subjects are adequately informed of the responsibilities and actions required when AEs occur. Trial subjects will be asked at each visit whether they have experienced AEs or SAEs. The subjects will additionally receive a patient’s diary to document any symptoms or medical occurrence between the visits. The investigator will check the diary at every visit. All AEs will be documented in detail in the trial subject’s medical records and in the CRF. The investigator is responsible for training and monitoring the members of the trial team with regard to the documentation and reporting of (S)AEs.

If an adverse event occurs, the subject must be observed regardless of any causal relationship to the investigational product. Observation continues until the symptoms have subsided, laboratory values have returned to baseline, and a plausible explanation has emerged, or until the subject’s death or termination of the trial for that subject.

The trial will be supervised by a DSMB. This Board will receive a list with the AEs that have occurred in the trial on a regular basis to decide on the continuation or modification of the trial. This allows identification and averting risks for the subjects at an early stage.

Every SAE that occurs must be reported *immediately* (within 24 h) to the Sponsor or the commissioned safety management). Exceptions are those SAEs that are defined in the protocol as SAEs that do not have to be reported immediately.

The reporting of SAEs for study patients is carried out within the following time periods:

- After the subject has been randomized and has received trial drug for the first time
- Until the end of study (week 130, v10)
- All serious adverse events (SAEs), adverse events leading to treatment discontinuation, adverse events of special interest (AESIs), adverse events of Grade ≥ 3 or severe intensity, adverse events considered related to the investigational medicinal product, and adverse events occurring in ≥ 5% of participants in either treatment group will be reported in trial publications.

### Frequency and plans for auditing trial conduct {23}

The Sponsor has the right to carry out audits at the trial centre and other facilities that may be involved in the study as part of quality assurance. The aim of audits is to check the validity, verifiability and completeness of the data and the credibility of the clinical trial as well as to check whether the patient's rights and safety are guaranteed. The Sponsor can therefore commission persons who are otherwise not involved in the clinical trial (auditors). They are allowed to inspect all study-related documents (in particular: study protocol, questionnaires, patient files, documentation of study medication, study-related correspondence).

The Sponsor and all participating trial sites support auditors and inspections by the competent authorities and allow, in this context, the persons charged with these duties access to the original documentation.

All persons conducting audits will keep all personal subject data and other trial data confidential.

### Plans for communicating important protocol amendments to relevant parties (e.g., trial participants, ethical committees) {25}

The annual safety report (ASR) will be compiled according to the corresponding ICH guideline E2F “Development Safety Update Report – DSUR” and SOP-PM-15 of DZNE e. V.

The start of the annual period for the ASR is the month and date of the “Development International Birth Date” (DIBD). The data lock point of the DSUR should be the last day of the one-year reporting period.

The data lock point for the patient data to be included and analyzed is defined by:

- The DIBD of the study drug

Alternatively:

- By the first approval of the clinical trial by a competent higher authority of a country within the EU in which the clinical trial is carried out

The Sponsor will supply the report within 60 days after the reference date (data lock point).

### Dissemination plans {31a}

It is intended to present the study protocol and later the study results within scientific talks, e.g., at the annual conference of the German Society of Neurology. Once the project is complete, data should be published in a peer-reviewed journal with open-access publishing option. Raw data and Excel sheets will be made available to the journal if possible. In the case of “negative” results data will be made available too in order to reduce future publication bias of other studies. We consider pre-publishing the results of the project on a public depository such as bioRxiv. By sharing the results through open-access and pre-publishing them once they are available, but also by publishing the study protocol before the actual start of the trial, we aim at reaching a broad community and especially other ALS specialists. Specialists from other university hospitals could be possible partners for a following confirmatory trial.

It is planned to present the results of the clinical trial in due time and after mutual coordination with the project leader in a scientific journal and/or at German and international congresses. In principle, preference should be given to a complete publication of the clinical trial. The “Uniform requirements for manuscripts submitted to biomedical journals (International Committee of Medical Journal Editors” (ICMJE) [JAMA 1997; 277: 927-34]), CONSORT (Consolidated Standards of Reporting Trials) Statement and the FAIR (Findable, Accessible, Interoperable, Reusable) Data Principle are taken into account.

The clinical trial is registered in the public register DRKS (German Clinical Trials Register) in accordance with the recommendations of the ICMJE.

## Discussion

ALS is a devastating disease leading to disability and death typically within 2–4 years after diagnosis. Rituximab is a medication that is frequently administered in autoimmune-mediated neurological diseases, where it proved to be efficient and safe. We anticipate a comparable safety profile in ALS, based on its tolerability in other neuroimmunological conditions, although this remains to be established in this population. Potential side effects of rituximab include infections, cytokine release syndrome (known in cancer patients), tumour lysis syndrome with acute renal failure, interstitial pneumonitis and severe infusion reactions such as drop in blood pressure or up to cardiac arrest. Many of these side effects are related to treatment trials in patients with myeloma, lymphoma and leukaemia who usually have high comorbidities and are therefore very different to neuroimmunological cohorts and patients in this study. Trials for neuroimmunological indications, such as in neuromyelitis optica (NMO), MS or encephalitis, show a much better safety profile. For example, no relevant side effects were observed in 30 patients with NMO with an average follow-up of 5 years [11]. Additionally, in patients tolerating i.v. rituximab infusions, rituximab can be regularly administered subcutaneously after individual consideration and in an outpatient clinical setting consistent with its established tolerability in these neuroimmunological indications (Mihajlović et al. [16]). ALS patients, which will be recruited for the proposed clinical trial here, will be restricted to patients with a maximal disease duration of 24 months after symptom onset. This will result in a study cohort with comparable to or with even less comorbidity than multiple sclerosis or encephalitis patients and thus in a tolerable safety profile [23]. Patients with ALS will need mechanical ventilation at some point of the disease, here, immunosuppression with rituximab might constitute a risk factor for an increased frequency of pulmonary tract infections. These infections are rare and can usually be treated with antibiotics. In order to minimize the risk of infectious complications, rituximab can only be given after exclusion of an ongoing local or systemic infection by patient history, clinical examination and broad safety laboratory, including white blood cell count, CRP and urine dipstick.

Intravenous infusion of rituximab will be performed by a study nurse and physician in order to assure correct administration and to detect and counteract potential near-term adverse effects immediately. These include infusion-related reactions (IRRs) with allergic reactions such as headache, itchiness, coughing, redness, exanthema, urticaria, hypertonia and fever. This can be controlled with reduction of infusion speed, antipyretics and antihistamines. Additionally, sodium chloride infusions, bronchodilatative medication and glucocorticoids will be administered in case of severe adverse events. If IRRs occur, infusion can be stopped immediately. Based on the trial protocol, regular clinical visits will be performed to assure patient safety and include monitoring of vital signs.

Together, the possible slight increase in infections by far exceeds the potential clinical benefit in this rapidly progressing disease. Based on the safety assessment and considered mechanism of action, the risk/benefit balance of this study is favorable.

This trial represents the first targeted evaluation of B-cell depletion therapy in ALS. Unlike previous immunosuppression trials using broad-spectrum agents (corticosteroids, cyclophosphamide, azathioprine) that lacked specificity and were associated with excessive side effects, rituximab offers specific targeting of the humoral arm of autoimmunity. Recent mechanistic evidence identifying pathogenic autoantibodies, oligoclonal B-cell expansions in CSF, and B-cell-dependent neuronal injury in preclinical models provides strong rationale for this targeted approach. By specifically depleting CD20+ B-cells while preserving other immune functions, rituximab may reduce autoimmune-mediated neuronal injury with an acceptable safety profile established in other neuroimmunological conditions.

This trial is explicitly designed as a mechanism-informed, signal-seeking phase-IIa study rather than a confirmatory efficacy trial. Its purpose is to establish biological plausibility, to assess whether B-cell depletion produces the expected biomarker modulation (sustained CD20+ B-cell depletion and changes in the autoantibody repertoire), and to evaluate feasibility, alongside exploratory clinical readouts. The clinical estimates from this pilot are therefore interpreted descriptively and for hypothesis-generating purposes, and are not powered to demonstrate a definitive disease-modifying effect. Accordingly, we caution against inferring a specific clinical effect size from this study; a favorable mechanistic and feasibility signal, even in the absence of statistically significant clinical benefit, would be considered an informative and encouraging result.

We further acknowledge that ALS is a clinically heterogeneous disease. Even within sporadic ALS of less than 24 months’ duration, disease trajectories vary substantially according to site of onset (bulbar versus limb), rate of functional decline and other prognostic factors. This heterogeneity can dilute an apparent treatment effect in a small cohort and complicate its interpretation. Site of onset and baseline progression rate are therefore recorded at baseline and, as described above, compared descriptively between groups and explored in sensitivity analyses; they are also candidate variables for the exploratory identification of potential responder subgroups that could inform stratification in a future confirmatory trial.

The eligibility criteria deliberately select a relatively preserved, early-stage ALS population (disease duration ≤ 24 months, slow vital capacity ≥ 75% predicted, no established need for assisted ventilation) in order to minimize infection and respiratory risk during immunosuppressive treatment. A consequence of this choice is that the findings may not extrapolate to later-stage ALS or to patients already requiring ventilatory support, and bulbar-onset disease was neither capped nor stratified at randomization. We regard site of onset as a potential effect modifier, and future trials may need stratified or stage-specific designs to establish efficacy across the full spectrum of ALS.

## Trial status

Protocol version number V9.0, 06.10.2025. The ABCD trial started recruitment in June 2024 and aims at completing recruitment by end of September 2026.

## Data Availability

The database of the trial is completely in the control of principial and coordinating investigator and sponsor representative.

## Abbreviations

AE: Adverse event
ALS: Amyotrophic Lateral Sclerosis
ALSFRS-R: ALS Functional Rating Scale - revised
AMG: Arzneimittelgesetz (German Drug Law)
aPTT: Activated partial thromboplastin time
AR: Adverse reaction
ASR: Annual safety report
BfArM: Bundesinstitut für Arzneimittel und Medizinprodukte
CA: Competent authority
CNS: Central nervous system
CONSORT: Consolidated Standards of Reporting Trials
CRF: Case report form
CSF: Cerebrospinal fluid
CTR: Regulation (EU) No 536/2014 of the European Parliament and of the Council
DMP: Data management plan
DSMB: Data and Safety Monitoring Board
DZNE: Deutsches Zentrum für Neurodegenerative Erkrankungen
ECAS: Edinburgh Cognitive and Behavioural ALS Screen
ECG: Electrocardiogram
eCRF: Electronic Case Report Form (Validated Study Database)
FAIR: Findable, Accessible, Interoperable, Reusable
i.v.: Intravenous
ICH-GCP: International Conference on Harmonisation of Technical Requirements for Registration of Pharmaceuticals for Human Use - Good Clinical Practice
IMP: Investigational medicinal oroduct
INR: International normalized ratio
ITT: Intention-to-treat
LPFV: Last patient first visit
LPLV: Last patient last visit
mITT: Modified intention-to-treat
MRI: Magnetic resonance imaging
PEI: Paul-Ehrlich-Institute
SAE: Serious adverse event
SDV: Source Data Verification
SOP: Standard operating procedure
SUSAR: Suspected, unexpected, serious adverse reactions
VC: Vital capacity

## References

[1] Atanasio A, Decman V, White D, Ramos M, Ikiz B, Lee H-C, et al. *C9orf72* ablation causes immune dysregulation characterized by leukocyte expansion, autoantibody production and glomerulonephropathy in mice. Sci Rep. 2016. 10.1038/srep23204. PMC4793236 26979938

[2] Castrillo-Viguera C, Grasso DL, Simpson E, Shefner J, Cudkowicz ME. Clinical significance in the change of decline in ALSFRS-R. Amyotroph Lateral Scler. 2010;11(1–2):178–80. 10.3109/17482960903093710 19634063

[3] Conti E, Sala G, Diamanti S, Casati M, Lunetta C, Gerardi F, et al. Serum naturally occurring anti-TDP-43 auto-antibodies are increased in amyotrophic lateral sclerosis. Sci Rep. 2021. 10.1038/s41598-021-81599-5. PMC7820419 33479441

[4] Cook CE. Clinimetrics corner: the minimal clinically important change score (MCID): a necessary pretense. J Man Manip Ther. 2008. 10.1179/jmt.2008.16.4.82E. PMC2716157 19771185

[5] Cui C, Longinetti E, Larsson H, Andersson J, Pawitan Y, Piehl F, Fang F. Associations between autoimmune diseases and amyotrophic lateral sclerosis: a register-based study. Amyotroph Lat Scler Frontotemp Degen. 2021;22(3-4):211–9. 10.1080/21678421.2020.1861022 33331190

[6] Engelhardt JI, Appel SH. IgG reactivity in the spinal cord and motor cortex in amyotrophic lateral sclerosis. Archiv Neurol. 1990;47(11):1210–6. 10.1001/archneur.1990.00530110068019 2122877

[7] Fournier CN, James V, Glass JD. Clinically meaningful change: evaluation of the Rasch-built overall amyotrophic lateral sclerosis disability scale (ROADS) and the ALSFRS-R. Amyotroph Lateral Scler Frontotemporal Degener. 2023;24(3–4):311–6. 10.1080/21678421.2022.2153607 36476010

[8] Hardiman O van den Berg LH, Kiernan M. Clinical diagnosis and management of amyotrophic lateral sclerosis. Nat Rev Neurol. 2011;7(11):639–49. 10.1038/nrneurol.2011.153 21989247

[9] Hauser SL, Waubant E, Arnold DL, Vollmer T, Antel J, Fox RJ, . . . HERMES Trial Group. B-cell depletion with rituximab in relapsing-remitting multiple sclerosis. The New Engl J Med. 2008; 358(7):676–88. 10.1056/NEJMoa0706383 18272891

[10] Kaufmann P, Levy G, Thompson JL, Delbene ML, Battista V, Gordon PH, . . . Mitsumoto H. The ALSFRSr predicts survival time in an ALS clinic population. Neurol. 2005;64(1):38–43. 10.1212/01.WNL.0000148648.38313.64 15642901

[11] Kim SH, Huh SY, Lee S, Joung A, Kim HJ. A 5-year follow-up of rituximab treatment in patients with neuromyelitis optica spectrum disorder. JAMA Neurol. 2013; 70(9):1110–7. 10.1001/jamaneurol.2013.3071 23897062

[12] Liu J, Wang F. Role of neuroinflammation in amyotrophic lateral sclerosis: cellular mechanisms and therapeutic implications. Front Immunol. 2017. 10.3389/fimmu.2017.01005. PMC5567007 28871262

[13] Mandrioli J, Biguzzi S, Guidi C, Sette E, Terlizzi E, Ravasio A, . . . D'Alessandro R. Heterogeneity in ALSFRS-R decline and survival: a population-based study in Italy. Neurological Sciences: Off J Ital Neurol Soc Ital Soc Clin Neurophysiol. 2015;36(12):2243–52. 10.1007/s10072-015-2343-6 26205535

[14] May C, Nordhoff E, Casjens S, Turewicz M, Eisenacher M, Gold R, . . . Meyer HE. Highly Immunoreactive IgG Antibodies Directed against a Set of Twenty Human Proteins in the Sera of Patients with Amyotrophic Lateral Sclerosis Identified by Protein Array. Plos One. 2014;9(2):e89596. 10.1371/journal.pone.0089596 PMC3935926 24586901

[15] McCombe PA, Henderson RD. The Role of immune and inflammatory mechanisms in ALS. Curr Molec Med. 2011;11(3):246–54. 10.2174/156652411795243450 PMC3182412 21375489

[16] Mihajlović J, Bax P, van Breugel E, Blommestein HM, Hoogendoorn M, Hospes W, Postma MJ. Microcosting Study of rituximab Subcutaneous Injection Versus Intravenous Infusion. Clin Therap. 2017;39(6):1221–32. 10.1016/j.clinthera.2017.05.342 28579210

[17] Mora JS, Genge A, Chio A, Estol CJ, Chaverri D, Hernández M, . . . AB10015 STUDY GROUP. Masitinib as an add-on therapy to riluzole in patients with amyotrophic lateral sclerosis: a randomized clinical trial. Amyotrop Late Scler Frontotemp Degen. 2024;25(1-2):5–14. 10.1080/21678421.2019.1632346 31280619

[18] Pullen AH, Demestre M, Howard RS, Orrell RW. Passive transfer of purified IgG from patients with amyotrophic lateral sclerosis to mice results in degeneration of motor neurons accompanied by Ca2+ enhancement. Acta Neuropathol. 2004;107(1):35–46. 10.1007/s00401-003-0777-z 14551798

[19] Schumacher H, Meyer T, Prüss H. GABAB receptor encephalitis in a patient diagnosed with amyotrophic lateral sclerosis. BMC Neurol. 2019. 10.1186/s12883-019-1269-7. PMC6416932 30871492

[20] Stud Working Group. G.-1. Safety and efficacy of edaravone in well defined patients with amyotrophic lateral sclerosis: a randomised, double-blind, placebo-controlled trial. The Lancet Neurol. 2017;16(7):505–12. 10.1016/S1474-4422(17)30115-1 28522181

[21] Uenal H, Rosenbohm A, Kufeldt J, Weydt P, Goder K, Ludolph A, et al. Incidence and geographical variation of amyotrophic lateral sclerosis (ALS) in southern Germany – completeness of the ALS registry Swabia. PLoS ONE. 2014. 10.1371/journal.pone.0093932. PMC3983245 24722455

[22] Van Damme P, Robberecht W, Van Den Bosch L. Modelling amyotrophic lateral sclerosis: progress and possibilities. Dis Model Mech. 2017;10(5):537–49. 10.1242/dmm.029058. PMID: 28468939; PMCID: PMC5451175. PMC5451175 28468939

[23] Westeneng HJ, Debray TP, Visser AE, van Eijk RP, Rooney JP, Calvo A, . . . Sommer H. Prognosis for patients with amyotrophic lateral sclerosis: development and validation of a personalised prediction model. Lancet Neurol. 2018;17(5):423-33. 10.1016/S1474-4422(18)30089-9 29598923

